# Natrum Mur. in Its Antidotal Sphere

**Published:** 1887-04

**Authors:** Wm. Jefferson Guernsey

**Affiliations:** Philadelphia


### NATRUM MUR. IN ITS ANTIDOTAL SPHERE.

WM. JEFFERSON GUERNSEY, M. D., PHILADELPHIA.

There hangs the proviso of some "if" about every condition of this world, and even those results which seem assured to us depend upon conditional circumstances. So, when about to prescribe an apparently well-indicated medicine, it is at least wise to consider if we have ascertained all that is discoverable about the case.

On the 7th of last September Mrs. S. P., æt. about thirty-two years, mother of two children, and a woman of excellent physique, came to me with a distressing throat trouble. I found the tonsils but slightly enlarged, a little inflamed, and accompanied by some catarrhal secretion. The chief annoyance was a sensation of swelling or "lump," which could not be swallowed, yet required constant effort to do so. Empty deglution troubled her more than swallowing either food or drink, yet the food seemed to lodge in the throat and then felt *as if passing over a sore spot.* I had never seen the lady before, but she informed me that she had used many gargles and been under allopathic treatment for this affection for six weeks without relief. She complained of absolutely no other trouble, though prostrated from loss of sleep from this. I thought I had an easy case, and gave a few doses of Bary. cb.$^{50m}$ (Sw.) Four days later she reported "no change whatever." Further questioning developed nothing new and I repeated the remedy in the same potency—one that I swear by. Her next report was as before, and I ventured with the Lach.$^{cm}$, then Bell.$^{cm}$, and still the same story. I noticed at the next visit that her lips were covered with fever blisters and called a halt. Here was a new departure. Not until this date had I followed an important injunction of our master. Part of § 207 of the *Organon* reads: "Inquire to what allopathic treatment the patient has been hitherto subjected—to determine the course to pursue to correct, if possible, this artificial deterioration." I accordingly questioned her closely about her former treatment but elicited nothing of importance. One more point, "Did you have any other ailment prior to this for which your allopathic physician had treated you?" Her answer was all that I could wish. She had had a severe ulceration of the womb and been "burnt out" several times, "but that was now well."(?) How soon after that trouble was "well" did this appear? She replied the very day that the discharge, which had been terribly profuse, had ceased (and suddenly), her throat had commenced to "choke." Did the allopath associate the two conditions? Not a bit of it. I could not ascertain with any degree of certainty what caustic had been used, but suspected the Nitrate of Silver from the fact that the herpetic eruption was strongly indicative of Nat. mur., its most powerful antidote. I accordingly gave six powders of Nat. mur.$^{295m}$ (F.) and told her not to be alarmed if the discharge from the vagina returned. Several days later she came into my office in great distress, exclaiming, "O Doctor! my ulceration has all come back. *What shall* I do!" "How about the throat?" "Oh! that is almost well, but the old discharge is as bad as ever it was. What *shall* I do?" I told her to go home and finish the medicine (Sac. lac.) and report. From that time on I had smooth sailing, and my case is now *well*, not palliated or suppressed, but *cured*. Doubtless the Bary. cb. would have acted nicely if the antidote had not been needed.

